# Expressed Alu repeats as a novel, reliable tool for normalization of real-time quantitative RT-PCR data

**DOI:** 10.1186/gb-2010-11-1-r9

**Published:** 2010-01-28

**Authors:** Manuela Marullo, Chiara Zuccato, Caterina Mariotti, Nayana Lahiri, Sarah J Tabrizi, Stefano Di Donato, Elena Cattaneo

**Affiliations:** 1 Department of Pharmacological Sciences and Center for Stem Cell Research, University of Milan, 9 Via Balzaretti, Milan, 20133, Italy; 2 Division of Biochemistry and Genetics, National Neurological Institute-IRCCS "Carlo Besta", 11 Via Celoria, Milan, 20133, Italy; 3 Department of Neurodegenerative Disease, UCL Institute of Neurology University College London and National Hospital for Neurology and Neurosurgery, Queen Square, London, WC1N 3BG, UK

## Abstract

We describe a novel strategy for mRNA normalization in quantitative real-time PCR that is based on expressed Alu repeat amplification as a measure for the mRNA fraction. We show that expressed Alu repeat amplification is a fast, accurate normalization tool that can be successfully used for quantification of selected mRNA in the human transcriptome. This result is particularly important for clinical diagnosis and biomarker validation studies based on mRNA detection in human blood.

## Abstract 2

Expressed Alu repeats are a reliable, accurate and universal reference for use in RT-qPCR normalization of human genes

## Background

Several factors hinder the reliable quantification of mRNA in gene expression studies performed in human samples, including sample availability, handling, and storage and the strategy used to normalize selected mRNA over the total mRNA fraction. With real-time quantitative RT-PCR (RT-qPCR), quantification errors depend on variations in the amount of total RNA or cDNA between samples, which remain difficult to determine. Various strategies have been applied in efforts to normalize these variations. Under controlled conditions of reproducible extraction of good-quality RNA, one possible strategy relies on total RNA quantification (RNA mass quantity) and/or the use of synthetic internal standards. To date, the amplification of a reference gene as the internal standard is the most frequently used approach to normalizing the mRNA fraction. This strategy is best applied when a large number of target genes are to be screened and makes inter-laboratory standardization easier than using total RNA content, which might depend on the quantification method. However, the reliable quantification of selected targets requires identification of a proper internal control gene for normalization, one that exhibits stable expression in the given cell or tissue under investigation.

A first accurate strategy for normalization was based on the GeNorm algorithm, which allows identification of the most stably expressed control genes in a human tissue of interest. The expression of ten common reference genes is analyzed in the tissue investigated by RT-qPCR, and the GeNorm algorithm is applied to calculate the gene expression stability (M) of the different control genes. The level of the target gene is normalized over the normalization factor (NF) obtained from the geometric mean of expression value of three or more stable reference genes [1].

Although the GeNorm algorithm is a robust normalization method, the reference gene validation requires extensive experimental work and a high quantity of sample material. These requirements may represent a problem in the context of gene expression studies involving the human transcriptome for which a limited amount of human tissue samples are available. Moreover, the choice of stable control genes becomes particularly difficult and is an expensive and lengthy process in pathological conditions characterized by transcriptional dysregulation. Altered activity of transcription factors and DNA target sequences may also affect the stability of mRNA expression levels of the selected reference gene, thus producing an erroneous quantification of the selected mRNA. This possibility leads to the requirement of selecting many genes and extensive experimental work before choosing a panel of stable reference genes.

To avoid these problems, we propose a new strategy for mRNA normalization in RT-qPCR that is based on expressed Alu repeat (EAR) amplification as a measure for the total mRNA fraction. More than one million copies of the approximately 300-bp Alu element are interspersed throughout the human genome, with up to 75% of all known genes containing Aluinsertions within their introns and/or untranslated regions (UTRs) [2]. Therefore, the differential expression of a number of genes in the tissues or cells under investigation will not influence EAR abundance in the transcriptome.

In this study, we set up a standardized RT-qPCR protocol for EAR amplification in the human transcriptome. We provide evidence that normalization based on EAR amplification is a suitable, fast, and precise tool for quantification of selected mRNA by RT-qPCR in human biological samples.

## Results

There is a long-standing interest in brain-derived neurotrophic factor (BDNF) because of its implications in neurodegenerative diseases [3]. BDNF is a neurotrophin that is crucial for the survival, maintenance, and differentiation of subpopulations of neurons in the central and peripheral nervous systems. Alterations in BDNF brain levels and activity have been described in various neurodegenerative disorders, most notably Huntington's disease (HD) [3]. Although BDNF is highly concentrated in the nervous system, it can also be detected in human blood and the blood of other mammals [4,5]. Because of that, several efforts have targeted testing BDNF levels in blood with the aim of assessing its potential value as a disease biomarker. For HD and other diseases, such as depression, schizophrenia, and Alzheimer's disease, its reliable detection in peripheral tissues may be crucial for understanding biological and pathogenic processes in humans and for assessing the potential activity of candidate therapeutic agents. Unfortunately, in human blood, this measurement has always proved problematic, especially for the BDNF protein. In fact, many factors influence this measurement, including the way the blood is collected, its storage, and the methodology employed to prepare the samples [6].

**Figure 1:**
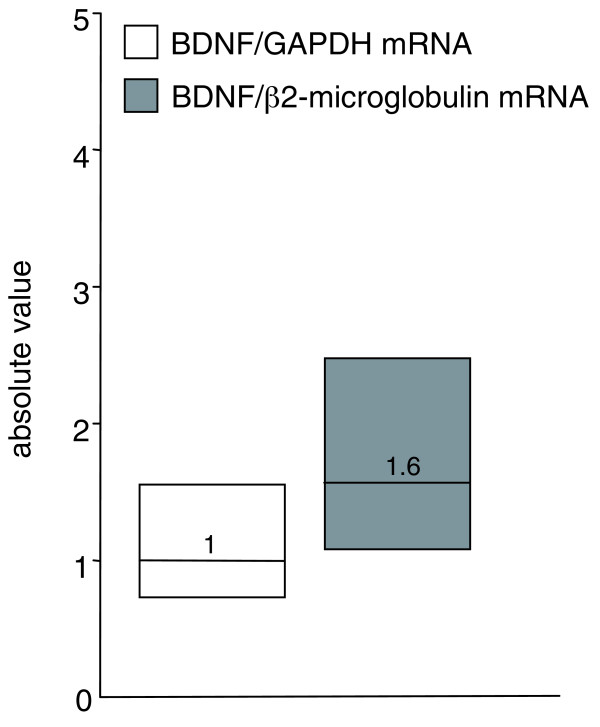
***BDNF *mRNA in human blood normalized to *GAPDH *and β*2M***. Median *BDNF *mRNA levels in the blood of eight control participants from the Institute of Neurology, MRC Prion Unit, and University College London (UK). The *BDNF *level was determined by means of quantitative real-time PCR and normalized to the level of *GAPDH *mRNA and β*2M*. The values are the averages of six independent PCR experiments. The boundary of the box closest to zero indicates the 25th percentile, the line within the box marks the median, and the boundary of the box farthest from zero indicates the 75th percentile.

When considering the measurement of blood BDNF mRNA, recovery and storage of samples are less problematic because the PAXgene™ Blood RNA System (PreAnalytiX, QIAGEN, Hilden, Germany) allows stabilization of intracellular RNA without affecting RNA isolation and yield. Given the remarkable reduction in brain BDNF mRNA levels in HD, we set up a fast, accurate, and standardized method for BDNF mRNA detection in blood. As for any other mRNA, this approach requires a normalization procedure that does not affect the experimental data. However, as Figure 1 shows, when the BDNF mRNA level in blood from eight control participants was normalized to glyceraldehyde-3-phosphate dehydrogenase (GAPDH) mRNA or β2-microglobulin (β2M) mRNA, the analyses produced different quantifications of BDNF mRNA levels normalized to these two different reference genes (median value BDNF mRNA/GAPDH mRNA, 1; median value BDNF mRNA/β2M mRNA, 1.6). These data support the notion that RT-qPCR performed with a conventional normalization strategy based on a single control gene may lead to erroneous quantification of target genes in human blood.

### Set up of a new normalization strategy: EAR amplification

**Figure 2:**
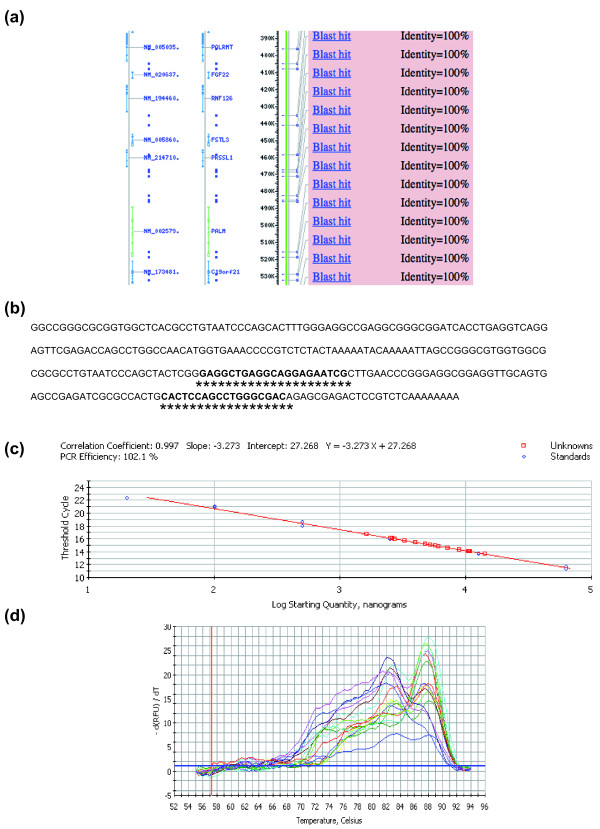
**EAR amplification protocol in RNA from total blood**. **(a) **BLAST analysis for Alu repeat distribution in the human genome. Alu repeat sequences are present in the UTRs of many genes. The small blue points indicate the homologous sequences at the Alu human consensus repeat. **(b) **Alu human interspersed repetitive sequence and primers used in this study for EAR amplification. **(c) **C_q _of EAR mRNA plotted against the log of the relative initial amount of the pooled cDNA. **(d) **Melt-curve diagram generated from the amplification as described in (c).

In the search for a stable tool for normalization, we focused on EARs because they are the most abundant repeats in the human genome. More than 1,500 human genes contain one or more Alu repeat in the UTRs regions. The EARs are about 280 nucleotides long and are divided into 30 well-conserved subfamilies (see [7] for a review). These features make EAR amplification a reliable tool for normalization in RT-qPCR based on the principle that the differential expression of a small number of genes bearing the EAR will not influence EAR abundance in the transcriptome. Figure 2a shows a BLAST representation of EAR element sequence conservation in the human genome. The small blue dots represent the position of EAR in the intergenic and intragenic regions and show that EARs are particularly located in the UTR and intronic regions. The Alu interspersed repetitive sequence and primers selected for the study are represented in Figure 2b while Additional file 1 shows the protocol for EAR amplification in the human transcriptome. As indicated, we first performed a retrotranscription (RT) reaction with total RNA at a final concentration of 12.5 ng/μl (see Materials and methods for additional details). The obtained cDNA was then diluted 1:200 and 5 μl of this solution tested using RT-qPCR (see Materials and methods). Notably, another advantage of this assay is in the low amount of cDNA used for the analyses, allowing many experimental replicates when the starting material (RNA) is limiting. As Figure 2c shows, we obtained a good RT-qPCR outcome, as indicated by the PCR efficiency (102%) and correct slope value (-3.27). In addition, another important outcome arises from the analysis of the amplified products with the melting curve. As Figure 2d shows, multiple melting peaks emerge from these analyses, and the expected peaks by amplified products (88,69 T) can be recognized. This result is anticipated given that several peaks are generated by the polymorphisms present in the EAR sequences and by different Alu subfamilies [7]. We conclude that when working with EARs, different melting curves in different samples are to be expected and that for each sample the replicates have to share their melting curve.

### Comparison of reference gene stability in human blood versus EARs

As indicated above, the use of NF obtained from the geometric mean of three or more stable reference genes is suitable for normalization [1]. We therefore decided to compare the EAR normalization tool to the normalization performed based on three more stable control genes expressed in blood and selected by the GeNorm algorithm. We started by choosing six different reference genes with expression in human blood that has been evaluated by RT-qPCR. The GeNorm algorithm was applied to calculate the M (see Table 1 for full gene name, accession number, chromosomal localization, indication that primers span an intron, and primer sequences). Genes with higher M values have greater variation in expression, and the threshold proposed for eliminating a gene as unstable was M ≥ 0.5.

**Table 1:** Primers sequences for GeNorm

Symbol	Name	NCBI accession number	Localization	Span intro	Sequences
*ACTB*	Beta actin	NC_001101	Chromosome 7	No	Fw:5'-AGTGTGACGTGGACATCCGCA-3'
					Rev:5'-GCCAGGGCAGTGATCTCCTTCT-3'
					
*HPRT1*	Hypoxanthine purine synthesis phosphoribosyl-transferase 1	NC_000023.9	Chromosome x	Yes	Fw:5'-ATGACCAGTCAACAGGGGACAT-3'
					Rev: 5'-CAACACTTCGTGGGGTCCTTTTCA-3'
					
*YWHAZ*	Tyrosine 3-monooxygenase/S tryptophan 5-monooxygenase activation protein, zeta polypeptide	NC_000008.9	Chromosome 8	Yes	Fw: 5'-CGTTACTTGGCTGAGGTTGCC-3'
					Rev: 5'-GTATGCTTGTTGTGACTGATCGAC-3'
					
*GNB2L1*	Guanine nucleotide binding protein, beta polypeptide 2-like 1	NC_000005.8	Chromosome 5	Yes	Fw: 5'-GAGTGTGGCCTTCTCCTCTG-3'
					Rev: 5'-GCTTGCAGTTAGCCAGGTTCC-3'
					
*B2M*	Beta-2-microglobulin	NC_000015.8	Chromosome 15	No	Fw: 5'-GGAGAGAGAATTGAAAAAGTGGAGC-3'
					Rev: 5'-GGCTGTGACAAAGTCACATGGTT-3'
					
*GAPDH*	Glyceraldehyde-3-oxidoreductase phosphate dehydrogenase	NC_000012.10	Chromosome 12	No	Fw: 5'-AGCTGAACGGGAAGCTCACT-3'
					Rev: 5'-AGGTCCACCACTGACACGTTG-3'

**Figure 3:**
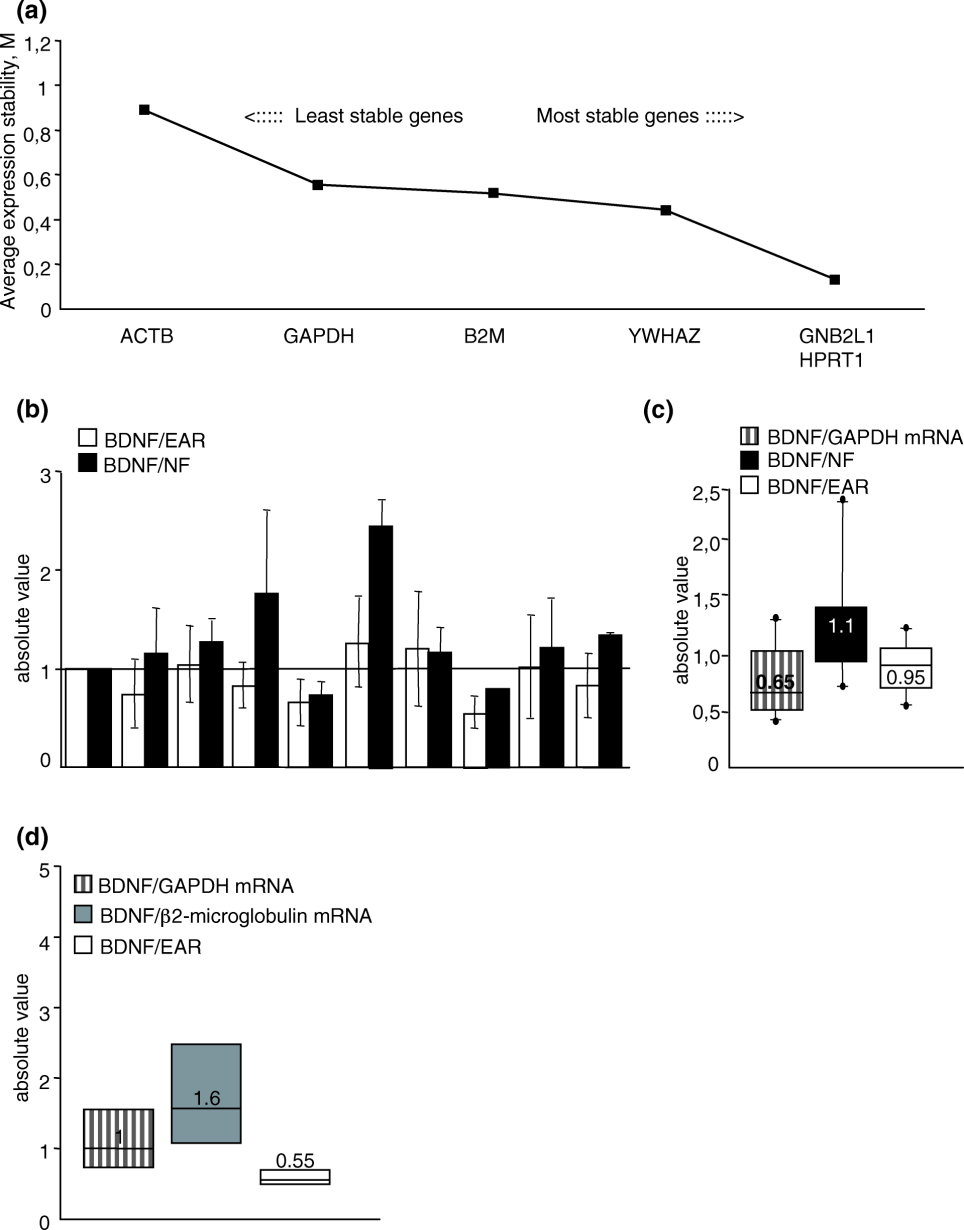
**Validation of EAR as a tool for normalization in RT-qPCR**. **(a) **Application of the GeNorm algorithm to eight genes to identify the three most stable housekeeping genes in blood. Average expression stability values (M) of remaining control genes during stepwise exclusion of the least stable control gene in the human blood of eight control participants. **(b) **Blood *BDNF *mRNA relative to EAR and to the three most stably expressed housekeeping genes. Pearson's correlation r = 0.56; *P *< 0.05. **(c) **Median blood *BDNF *mRNA levels versus *GAPDH*, versus the three most stable control genes and versus EAR in ten control participants. Samples used in (a-c) were from the National Neurological Institute-IRCCS "Carlo Besta", Milan (Italy). **(d) **Median blood *BDNF *mRNA levels versus *GAPDH*, versus *β2M*, and versus EAR mRNA in eight control participants from the Institute of Neurology, MRC Prion Unit, University College London (UK).

On the basis of our analyses, the ranking of the expression stability in these genes was (from most to least stable): *GNB2L1 *(guanine nucleotide binding protein (G protein), beta polypeptide 2-like 1), *HPRT1 *(hypoxanthine phosphoribosyltransferase 1), *YWHAZ *(tyrosine 3-monooxygenase/tryptophan 5-monooxygenase activation protein, zeta polypeptide), β*2M*, *GAPDH*, and β*ACT *(β-actin) (Figure 3a). As shown, the M values of *GNB2L1*, *HPRT1*, and *YWHAZ *were lower than 0.5; therefore, these genes were concluded to be stably expressed reference genes in human blood. The NF value, used for normalization of RT-qPCR, was calculated based on the geometric mean of the expression levels of these three most stable control genes.

To assess the validity of EARs as a normalization tool versus the GeNorm strategy, we evaluated BDNF mRNA levels normalized to EARs and to NF in blood from ten healthy volunteers. Figure 3b shows that the quantification of BDNF mRNA in human blood is comparable after normalization with the two different strategies. The median value shown in Figure 3c confirms this result: the median values of blood BDNF mRNA levels normalized versus EARs and versus NF are similar to each other (0.95 versus 1.1), while the median value of BDNF mRNA normalized versus GAPDH mRNA is much lower (0.65). These data indicate that EARs represent a good tool for normalization and that normalization over a single reference gene provides incorrect results.

Subsequently, we applied the EAR normalization tool for the quantification of BDNF mRNA in the eight control participants analyzed in Figure 1, in which the messenger level of the neurotrophin is represented relative to *GAPDH *or *β2M *mRNA content. Figure 3d shows the median value of *BDNF *mRNA normalized over *GAPDH *(1), *β2M *(1.6), or EARs (0.55). As shown, the distribution of the *BDNF *mRNA values normalized to EARs is tighter with respect to the distribution of *BDNF *mRNA normalized to *GAPDH *or *β2M *mRNA, indicating that the EAR normalization strategy removes most of the non-specific variations arising from the use of an inappropriate internal reference gene.

The experiments described above prove the reliability of the EAR normalization strategy with respect to GeNorm as well as the efficiency of this new method with respect to normalization to a single housekeeping gene to measure *BDNF *mRNA levels in human blood.

### EAR normalization applies to the quantification of mRNAs specifically expressed in blood

To evaluate the reliability of the EAR normalization strategy with other, more blood-specific genes, we analyzed *IL8 *(interleukin-8) mRNA levels in blood from control participants. Validation of EAR normalization for cytokine quantification in blood might be particularly useful for measuring the balance between pro- and anti-inflammatory cytokines in chronic inflammatory disorders or autoimmune disease. Cytokine mRNA quantification is therefore of clinical relevance to immuno-monitoring and for the evaluation of the disease status in patients.

**Figure 4:**
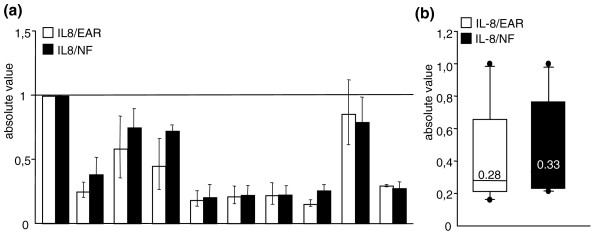
**Application of the EAR normalization tool for the amplification of a specific blood mRNA (*IL8*)**. **(a) **Amplification of blood *IL8 *mRNA relative to EAR mRNA and to the three most stably expressed housekeeping genes. Pearson's correlation r = 0.95; *P *< 0.0001. **(b) **Median blood *IL8 *mRNA levels versus EAR and versus the three most stable control genes in ten control participants. Samples from the National Neurological Institute-IRCCS "Carlo Besta", Milan (Italy).

In our measurements of *IL8 *mRNA levels in ten control participants, normalized versus EARs and versus NF, the *IL8 *mRNA levels with the two different approaches were similar in single human samples; also, again, the similar median value obtained (Figure 4b) confirmed the result.

We concluded that EARs are a suitable normalization tool for gene expression study of cytokines in human blood.

### EAR normalization for human tissues other than blood

Fast, sensitive, and accurate quantification in RT-qPCR analysis is a crucial tool not only for assessing mRNA in human blood but also for evaluating the levels of given molecules in tissues such as post-mortem human brain. In a previous study, we analyzed *BDNF *mRNA levels in 38 post-mortem human cortices from control participants and HD patients using *GAPDH *as the reference gene [8]. The *GAPDH *gene is, in fact, one of the more commonly used reference genes in such analyses [9].

**Figure 5:**
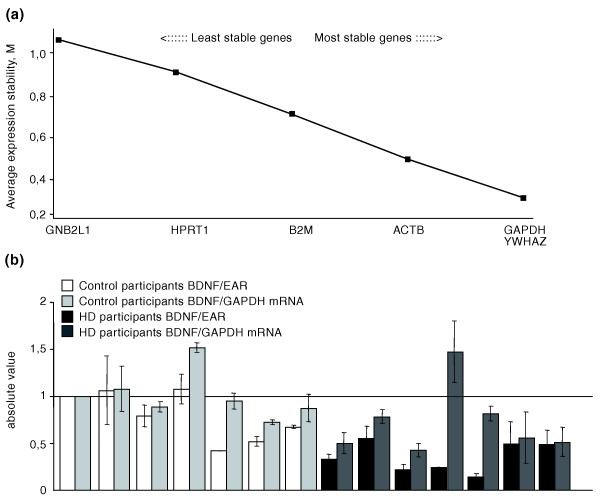
***BDNF *mRNA level normalized to EAR in the human post-mortem cortex (a) Application of the GeNorm algorithm to six genes to identify the three most stable housekeeping genes in human post-mortem cortex**. Average expression stability values (M) of remaining control genes during stepwise exclusion of the least stable control gene in the human cortex of seven control participants. **(b) ***BDNF *mRNA levels in the human cortex of seven control participants and seven HD patients normalized to *GAPDH *(Zuccato *et al*. [8]) and EAR mRNA.

Here we tested whether *GAPDH *is a stable control gene for brain tissues versus EAR and whether normalization over EAR still confirms the finding of a robust reduction in *BDNF *mRNA in HD cortical samples over controls [8]. To evaluate the efficiency of *GAPDH*, we used the same RNA as in Zuccato *et al*. [8] and performed a GeNorm analysis on six different reference genes. As shown in Figure 5a, the ranking of the expression stability in these genes was (from most to least stable): *GAPDH*, *YWHAZ*, *βACT*, *β2M*, *HPRT1*, and *GNB2L1*. The M values of *GAPDH *were lower than 0.5, confirming that this gene is a stably expressed reference gene in human cortex and also validating the previously performed quantification of the *BDNF *mRNA in human HD samples versus controls [8]. Using the same RNA, we next repeated the analysis of *BDNF *mRNA levels and used normalization to EARs or to *GAPDH*. Figure 5b shows that the *BDNF *mRNA level was lower in HD patients with respect to control participants (Mann-Whitney, *P *< 0.05, two-tailed test). These data demonstrate the efficiency of EAR normalization for quantifying a transcript of interest in other human tissues, such as cerebral cortex, that are normally subjected to a long post-mortem delay.

**Figure 6:**
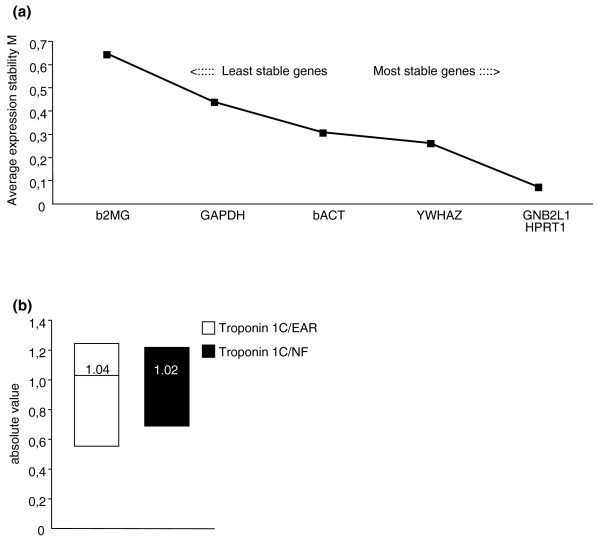
**Normalization to EAR and GeNorm to evaluate *TNNC1 *mRNA levels, a gene specifically expressed in human skeletal muscle**. **(a) **Application of the GeNorm algorithm to six genes to identify the three most stable housekeeping genes in human skeletal muscle. Average expression stability values (M) of remaining control genes during stepwise exclusion of the least stable control gene in the human skeletal muscle of four control participants. **(b) **Median skeletal muscle *TNNC1 *mRNA levels versus EAR and versus the three most stable control genes in four control participants. Samples from the National Neurological Institute-IRCCS "Carlo Besta", Milan (Italy). Pearson's correlation r = 0.94; *P *< 0.05

Moreover, we extended the application of EAR normalization strategy to samples from skeletal muscle, an accessible tissue that responds to hormonal, metabolic and neural inputs. The muscle, therefore, is a reasonable tissue to examine for gene expression changes that might be turned into biomarkers for neurodegenerative diseases [10]. In particular, we have analyzed the expression levels of *TNN1C *(troponin C type 1) mRNA, a stable gene expressed in the skeletal muscle. The GeNorm algorithm was applied to evaluate three more stable control genes expressed in human muscle. On the basis of our analyses, the ranking of the expression stability in these genes was (from most to least stable): *GNB2L1*, *HPRT1*, *YWHAZ*, β*2M*, *GAPDH*, and β*ACT *(Figure 6a). As shown, the M values of *GNB2L1*, *HPRT1*, and *YWHAZ *were lower than 0.5; therefore, these genes were concluded to be stably expressed reference genes in human skeletal muscle. The NF value, used for normalization of RT-qPCR, was calculated based on the geometric mean of the expression levels of these three most stable control genes. Importantly, in our measurements of *TNN1C *mRNA levels in four control participants, normalized versus EARs and versus NF, the *TNN1C *mRNA levels with the two different approaches were overlapping in single human samples. Figure 6b also shows a similar median value between the two measurements.

The data described above show that, in addition to peripheral blood, the EAR normalization strategy can be applied to samples from human brain and skeletal muscle, implying that this method could be used for assessing different human samples.

## Discussion

Accurate normalization of mRNA levels is an absolute prerequisite for obtaining reliable results in gene expression studies. Recently, considerable attention has focused on the systematic study of normalization procedures. Up to the year 2000, a number of articles discussed the problem of normalizing mRNA values over a reference gene, indicating that the most commonly used reference genes, such as *GAPDH *and β*ACT*, exhibit variation under different experimental conditions [9,11,12]. In addition, finding suitable reference genes is not straightforward, and different internal control genes should be tested in the context of a specific experimental design. Different groups have faced this problem [13,14], and interesting strategies have been proposed. In particular, Vandesompele *et al*. [1] elucidated the errors related to the common practice of single control normalization. The results showed that a conventional normalization strategy based on a single reference gene leads to erroneous normalization and also demonstrated that ideal and universal control genes do not exist. A proposed alternative strategy is based on a gene stability measure (M) as the average pair-wise variation between a particular gene and all other control genes. This approach has led to the GeNorm algorithm, which evaluates the M of common reference genes and allows determination of the most stably expressed genes in the tissue analyzed during stepwise exclusion of the worst scoring gene points [1]. The rationale for this analysis is that a normalization factor based on proper internal control genes should remove all non-specific variation. This robust procedure has led to more accurate gene expression studies; however, the method must be set up for different tissues analyzed and requires a high number of replicates, involving extensive experimental work and twice the amount of sample material. Despite being an important development, the GeNorm strategy does not completely satisfy the need for a universal method for normalization in RT-qPCR.

We therefore focused on the EARs. The location of EARs in UTRs of 1,500 genes makes the development of this novel strategy an important tool for normalization. In fact, even the transcriptional dysregulation that characterizes several pathological conditions does not significantly alter the total amount of EARs in the whole human transcriptome. This scenario applies in HD, in which the activity of several transcription factors and related DNA target sequences is impaired [15], thus specifically complicating the choice of a suitably stable reference gene both in the control and HD population.

One of the selected mRNA targets in this study, *BDNF*, is reported to be involved in the pathogenesis of HD [3,16]. Previous studies have shown that *BDNF *mRNA is consistently reduced in cells and *in vivo *brain tissues from HD transgenic mice and patients [3]. In addition, blood BDNF levels correlate with brain BDNF levels and disease progression in HD mice [17]. The protocol described here for the rapid and accurate evaluation of *BDNF *mRNA in human blood samples represents a step forward in efforts to develop a reliable measurement of key mRNA in blood. Moreover, we clearly demonstrated that mRNA levels normalized over EARs are comparable to those obtained after normalization using the most robust normalization strategies available in both analyzed tissues.

More important, given the widespread distribution of EARs in the human genome and the absence of any tissue specificity in their expression, the EAR normalization tool described here can be universally applied to human biological samples (blood, skeletal muscle and post-mortem brain tissues), avoiding the time-consuming and expensive experimental work needed for selection and analysis of the most stable reference genes in the chosen tissue. It should be noted that the EAR normalization strategy minimizes the amount of RNA used for the analysis, an important advantage for expression studies on samples characterized by a limited amount of RNA where it would otherwise be very difficult to use alternative methods such as GeNorm.

## Conclusions

A reliable and flexible normalization strategy has been an urgent need in the context of gene expression studies in human samples. We have identified a suitable novel normalization strategy that can be applied in different types of human biological material. EAR normalization enables the validation of an appropriate control gene for any specific experimental design to be avoided. More important, the strategy of reducing non-specific variation in samples by normalizing to EAR enables a straightforward assessment of relevant biological variation from mRNA levels. This possibility is particularly important in the context of clinical studies for evaluating the alteration of mRNA levels as reliable markers of disease progression.

## Materials and methods

### Recruitment of healthy volunteers

Individuals were recruited for the study from the National Hospital for Neurology and Neurosurgery, London (UK) and the National Neurological Institute-IRCCS "Carlo Besta", Milan (Italy). Full ethical review and consent was obtained for the research use of blood and skeletal muscle from healthy volunteers and all subjects gave informed written consent. Table 2 gives participant demographic characteristics.

**Table 2:** Characteristics of the study

	Number	Gender (F/M)	Age (years)
Blood			
University College London (UK)	8	4/4	44 (28-62)
Institute-IRCCS "Carlo Besta" (Italy)	10	7/3	31 (23-45)
			
Skeletal muscle			
Institute-IRCCS "Carlo Besta" (Italy)	4	2/2	35 (24-46)

F: female; M: male

### Human tissue collection

#### Blood collection

A total of 2.5 ml whole venous blood was collected in an evacuated blood collection tube (PAXgene™ Blood RNA Tube, BD Vacutainer™, Plymouth, UK) containing a stabilizing additive. After sample collection, the PAX tubes were incubated and stabilized at room temperature for 2 hours, followed by immediate storage at -20°C until RNA extraction.

#### Skeletal muscle collection

Biopsies of vastus lateralis muscle were obtained after informed consent from National Neurological Institute-IRCCS "Carlo Besta" (Italy). Biopsies were obtained under local anesthesia and immediately frozen in dry ice or liquid nitrogen.

### RNA extraction

RNA was extracted from whole blood using the PAXgene™ Blood RNA System Kit (PreAnalytiX, QIAGEN) following the manufacturer's guidelines. Total RNA concentration and purity were measured using a NanoDrop 1000 Spectrophotometer (Thermo Scientific, Waltham, MA, USA), and quality was verified by means of agarose gel electrophoresis of 1 μg of each sample (Additional data file 2). The total RNA was stored in aliquots at -80°C.

The muscle samples were homogenized in TRIZOL (Invitrogen, Carlsbad, CA, USA) using a Ultraturex homogeniser. Total RNA was isolated according to the manufacturer's protocol and stored in aliquots at -80°C.

### RNA retrotranscription

Using Superscript III RNaseH reverse transcriptase (Invitrogen) and random primers in a volume of 20 μl, 250 ng total RNA was reverse-transcribed to single-stranded cDNA, according to the manufacturer's instructions.

### Real-time PCR

Three independent PCR analyses were performed of each of two independent RT reactions, for a total of six independent measurements for each of the analyzed mRNA samples.

#### BDNF, IL8, TNNC1 and housekeeping genes

Using an iCycler Thermal Cycler with a Multicolor Real-time PCR Detection System (Bio-Rad, Hercules, CA, USA), all of the reactions were performed in a total volume of 25 μl containing 5 μl of cDNA from a 1:10 RT dilution, 50 mM KCl, 20 mM Tris-HCI, pH 8.4, 0.2 mM dNTPs, iTaq DNA polymerase, 25 units/ml, 3 mM MgCl_2_, SYBR Green I, 10 nM fluorescein, stabilizers (iQTM SYBR Green Supermix, Bio-Rad), and 0.3 μM of forward and reverse primers. The amplification cycles consisted of an initial denaturing cycle at 95°C for 3 minutes, followed by 45 cycles of 30 s at 95°C, 30 s at 60°C, and 30 s at 72°C. Fluorescence was quantified during the 60°C annealing step, and product formation was confirmed by means of a melting curve analysis (55 to 94°C). The amounts of *BDNF *and *IL8 *mRNA were normalized to the geometric mean of the three most stable housekeeping genes (*HPRT1*, *YWHAZ*, and *GNB2L*; Table 1 for primer sequences).

The *BDNF*-specific primers set up on the *BDNF *coding sequence (GenBank accession number AF411339) were *BDNF *5'-TAACGGCGGCAGACAAAAAGA-3' and *BDNF *5'-GAAGTATTGCTTCAGTTGGCCT-3'. The obtained amplification product was 101 bp long. The *IL8*-specific primers were set up referring to GenBank accession number NM_000584.2; the primer sequences were *IL8 *5'-CCATCTCACTGTGTGTAAACATGAC-3' and *IL8 *5'-TCCACTCTCAATCACTCTCAGTTCT-3'. The obtained amplification product was 194 bp long. The *TNNC1*-specific primers were set up referring to GenBank accession number NM_003280.2; the primer sequences were *TNNC1 *5'-TGCAGGAGATGATCGATGAGGTG-3' and *TNNC1 *5'-TGCGGAAGAGGTCAGACAGCTC-3'. The obtained amplification product was 138 bp long.

#### EARs

Using an iCycler Thermal Cycler with a Multicolor Real-time PCR Detection System (Bio-Rad), all of the reactions were performed in a total volume of 25 μl containing 5 μl of cDNA from a 1:200 RT dilution, 50 mM KCl, 20 mM Tris-HCI, pH 8.4, 0.2 mM dNTPs, iTaq DNA polymerase, 25 units/ml, 3 mM MgCl_2_, SYBR Green I, 10 nM fluorescein, stabilizers (iQTM SYBR Green Supermix, Bio-Rad), and 0.3 μM of forward and reverse primers. The amplification cycles consisted of an initial denaturing cycle at 95°C for 3 minutes, followed by 45 cycles of 30 s at 95°C, 30 s at 60°C, and 30 s at 72°C. Fluorescence was quantified during the 60°C annealing step, and product formation was confirmed by means of a melting curve analysis (55 to 94°C).

The EAR-specific primers were set up on an Alu human interspersed repetitive sequence (Figure 2b). The primer sequences were EAR 5'-GAGGCTGAGGCAGGAGAATCG-3' and EAR 5'-GTCGCCCAGGCTGGAGTG-3'. The obtained amplification product was 87 bp long.

### Statistical analyses and data representation

Because BDNF values did not have a normal distribution, the non-parametric Mann-Whitney two-tailed U-test was used for the statistical analyses, with significance set at *P *< 0.05. Median values were chosen because of the skewed distribution of the data. In each figure, the boundary of the box closest to zero indicates the 25th percentile, the line within the box marks the median or 50th percentile, and the boundary of the box farthest from zero indicates the 75th percentile. When ten or more samples were analyzed, whiskers above and below the box indicate the 90th and 10th percentiles.

The validation of the EAR normalization strategy with respect to the GeNorm algorithm was measured using Pearson correlations (r).

The materials and methods section and nomenclature follows the MIQE reporting guidelines [18].

## Abbreviations

β2M: β2-microglobulin; βACT: β-actin; BDNF: brain-derived neurotrophic factor; EAR: expressed Alu repeat; GAPDH: glyceraldehyde-3-phosphate dehydrogenase; GNB2L1: guanine nucleotide binding protein (G protein), beta polypeptide 2-like 1; HD: Huntington's disease; HPRT1: hypoxanthine phosphoribosyltransferase 1; IL: interleukin; M: gene expression stability measure; NF: normalization factor; RT: retrotranscription; RT-qPCR: real-time quantitative RT-PCR; TNNC1: troponin C type 1; UTR: untranslated region; YWHAZ: tyrosine 3-monooxygenase/tryptophan 5-monooxygenase activation protein, zeta polypeptide.

## Authors' contributions

MM, CZ, and EC conceived the project and wrote the manuscript. MM carried out the molecular biology experiments. MM, CZ, and EC have evaluated the results and interpreted the data. CM, SD, NL and ST provided blood samples for the analyses and evaluated the data. All authors approved the final manuscript.

## Supplementary Material

Additional file 1 Protocol for EAR amplification in the human transcriptome. Click here for file

Additional file 2 A selection of RNA from peripheral blood used in this study. Click here for file

## References

[B1] Vandesompele J De Preter K Pattyn F Poppe B Van Roy N De Paepe A Speleman F Accurate normalization of real-time quantitative RT-PCR data by geometric averaging of multiple internal control genes. Genome Biol 2002 3 RESEARCH0034.1 RESEARCH0034.11 10.1186/gb-2002-3-7-research0034 PMC126239 12184808

[B2] Yulug IG Yulug A Fisher EM The frequency and position of Alu repeats in cDNAs, as determined by database searching. Genomics 1995 27 544 548 10.1006/geno.1995.1090 7558040

[B3] Zuccato C Cattaneo E Brain-derived neurotrophic factor in neurodegenerative diseases. Nat Rev Neurol 2009 5 311 322 10.1038/nrneurol.2009.54 19498435

[B4] Fujimura H Altar CA Chen R Nakamura T Nakahashi T Kambayashi J Sun B Tandon NN Brain-derived neurotrophic factor is stored in human platelets and released by agonist stimulation. Thromb Haemost 2002 87 728 734 12008958

[B5] Radka SF Holst PA Fritsche M Altar CA Presence of brain-derived neurotrophic factor in brain and human and rat but not mouse serum detected by a sensitive and specific immunoassay. Brain Res 1996 709 122 130 10.1016/0006-8993(95)01321-0 8869564

[B6] Trajkovska V Marcussen AB Vinberg M Hartvig P Aznar S Knudsen GM Measurements of brain-derived neurotrophic factor: methodological aspects and demographical data. Brain Res Bull 2007 73 143 149 10.1016/j.brainresbull.2007.03.009 17499648

[B7] Batzer MA Deininger PL Alu repeats and human genomic diversity. Nat Rev Genet 2002 3 370 379 10.1038/nrg798 11988762

[B8] Zuccato C Marullo M Conforti P MacDonald ME Tartari M Cattaneo E Systematic assessment of BDNF and its receptor levels in human cortices affected by Huntington's disease. Brain Pathol 2008 18 225 238 10.1111/j.1750-3639.2007.00111.x 18093249 PMC8095509

[B9] Suzuki T Higgins PJ Crawford DR Control selection for RNA quantitation. Biotechniques 2000 29 332 337 10948434 10.2144/00292rv02

[B10] Strand AD Aragaki AK Shaw D Bird T Holton J Turner C Tapscott SJ Tabrizi SJ Schapira AH Kooperberg C Olson JM Gene expression in Huntington's disease skeletal muscle: a potential biomarker. Hum Mol Genet 2005 14 1863 1876 10.1093/hmg/ddi192 15888475

[B11] Bustin SA Absolute quantification of mRNA using real-time reverse transcription polymerase chain reaction assays. J Mol Endocrinol 2000 25 169 193 10.1677/jme.0.0250169 11013345

[B12] Bustin SA Quantification of mRNA using real-time reverse transcription PCR (RT-PCR): trends and problems. J Mol Endocrinol 2002 29 23 39 10.1677/jme.0.0290023 12200227

[B13] Overbergh L Giulietti A Valckx D Decallonne R Bouillon R Mathieu C The use of real-time reverse transcriptase PCR for the quantification of cytokine gene expression. J Biomol Tech 2003 14 33 43 12901609 PMC2279895

[B14] Pachot A Blond JL Mougin B Miossec P Peptidylpropyl isomerase B (PPIB): a suitable reference gene for mRNA quantification in peripheral whole blood. J Biotechnol 2004 114 121 124 10.1016/j.jbiotec.2004.07.001 15464605

[B15] Cha JH Transcriptional signatures in Huntington's disease. Prog Neurobiol 2007 83 228 248 17467140 10.1016/j.pneurobio.2007.03.004 PMC2449822

[B16] Zuccato C Cattaneo E Role of brain-derived neurotrophic factor in Huntington's disease. Prog Neurobiol 2007 81 294 330 10.1016/j.pneurobio.2007.01.003 17379385

[B17] Conforti P Ramos C Apostol BL Simmons DA Nguyen HP Riess O Thompson LM Zuccato C Cattaneo E Blood level of brain-derived neurotrophic factor mRNA is progressively reduced in rodent models of Huntington's disease: restoration by the neuroprotective compound CEP-1347. Mol Cell Neurosci 2008 39 1 7 10.1016/j.mcn.2008.04.012 18571429

[B18] Bustin SA Benes V Garson JA Hellemans J Huggett J Kubista M Mueller R Nolan T Pfaffl MW Shipley GL Vandesompele J Wittwer CT The MIQE guidelines: minimum information for publication of quantitative real-time PCR experiments. Clin Chem 2009 55 611 622 10.1373/clinchem.2008.112797 19246619

