# Supplementary figures and images for: Expressed Alu repeats as a novel, reliable tool for normalization of real-time quantitative RT-PCR data
Source: Genome Biol. 2010 Jan 28;11(1):R9. doi: 10.1186/gb-2010-11-1-r9 (PMC2847721; doi:10.1186/gb-2010-11-1-r9)

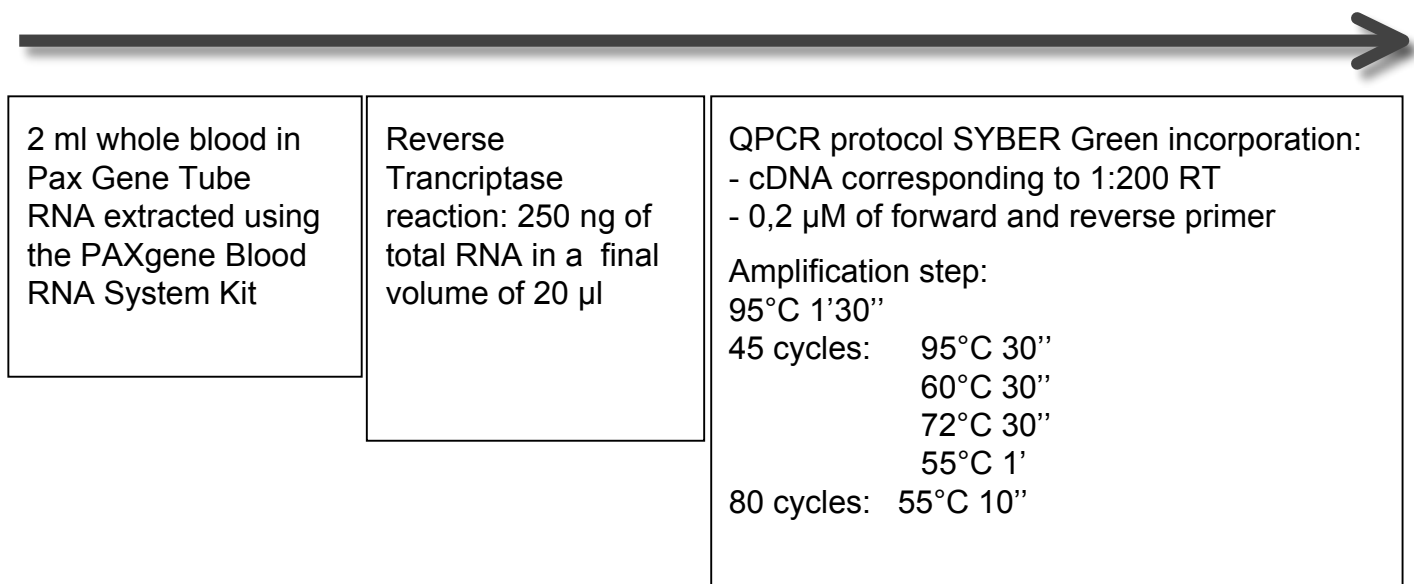

Supplement: Additional file 1 — Protocol for EAR amplification in the human transcriptome. [file gb-2010-11-1-r9-S1.pdf]

### A selection of RNA from peripheral blood

---

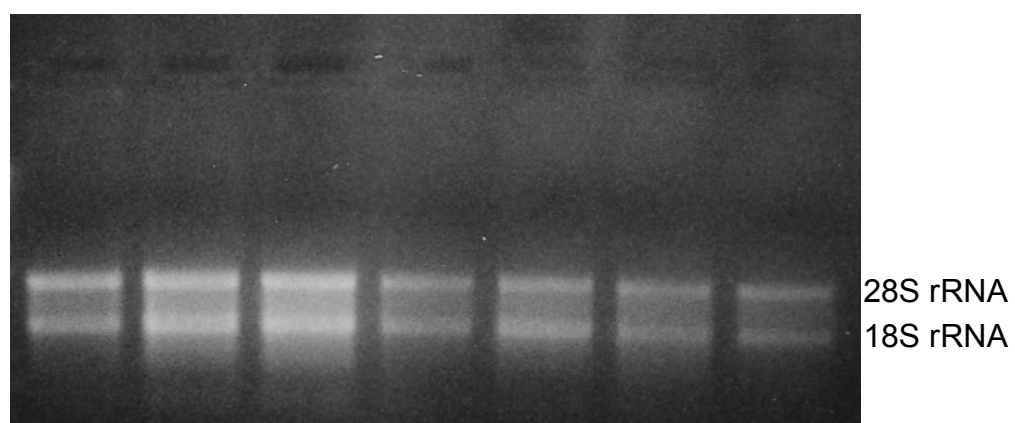

Supplement: Additional file 2 — A selection of RNA from peripheral blood used in this study. [file gb-2010-11-1-r9-S2.pdf]
